# Hypertrophic osteoarthropathy mimicking a reactive arthritis: a case report and review of the literature

**DOI:** 10.1186/s12891-018-2068-9

**Published:** 2018-05-14

**Authors:** Francesco Bozzao, Stella Bernardi, Franca Dore, Lorenzo Zandonà, Fabio Fischetti

**Affiliations:** 1 Department of Medical Sciences, University of Trieste, Cattinara Teaching Hospital, Strada di Fiume 449, 34149 Trieste, Italy; 2 ASUITS, Cattinara Teaching Hospital, Strada di Fiume 449, 34149 Trieste, Italy

**Keywords:** Hypertrophic osteoarthropathy, Reactive arthritis, Lepidic predominant lung adenocarcinoma, Periosteal reaction

## Abstract

**Background:**

Hypertrophic osteoarthropathy (HOA) is a syndrome characterized by abnormal proliferation of skin and periosteal tissues of the extremities. It can be a rare hereditary disease (pachydermoperiostosis) or can be secondary to various diseases, though mostly lung malignancies. Here, we report an unusual clinical presentation of HOA.

**Case presentation:**

A 77-year-old man presented with fever, diarrhea, and an oligoarthritis involving the left knee and the ankles. Since left knee synovial fluid aspiration revealed an aseptic synovitis and *Clostridium Difficile* toxin was detectable in stool samples, a reactive arthritis secondary to a *Clostridium Difficile* induced colitis was initially suspected. However, the presence of a worsened digital clubbing and the lack of a good clinical response to steroid therapy led us to perform a radionuclide bone scanning, which revealed HOA. This turned out to be associated with a lepidic predominant lung adenocarcinoma, which was clinically and radiologically difficult to distinguish from a relapse of pneumonia.

**Conclusion:**

Consistent with the literature, HOA tends to have a variable clinical presentation, mimicking that of various rheumatic diseases. This clinical case shows that HOA can present as a presumptive acute reactive arthritis, and it highlights the importance of patient’s follow-up in the differential diagnosis of inflammatory arthritis, especially when a worsened digital clubbing is present.

## Background

Rheumatic paraneoplastic syndromes include musculoskeletal disorders not directly caused by tumor expansion, but related with humoral factors released from deranged cells. These syndromes are rare and challenging to diagnose. Nevertheless, their prompt recognition can be of major clinical importance because they often precede other manifestations of the associated neoplasm [1]. Hypertrophic osteoarthropathy (HOA) can be a primary hereditary disease, but it is most often a rheumatic paraneoplastic syndrome secondary to a lung malignancy [2]. It is characterized by digital clubbing and periostosis of tubular bones [1]. Here, we report the case of a 77-year-old man presenting with an asymmetric oligoarthritis of the lower limbs, which was initially interpreted as a reactive arthritis associated with a *Clostridium Difficile* infection, but later understood to be a HOA associated with a lepidic predominant lung adenocarcinoma. We also performed a review of the literature and a search in Pubmed of other clinical cases of adult patients affected by primary or secondary HOA, in which other rheumatic diseases were initially suspected. For this purpose, we used the combined terms “hypertrophic osteoarthropathy” and “case report” and we selected only English written articles (Table 1).

**Table 1:** Rheumatic disorders mimicking and/or associated with hypertrophic osteoarthropathy

Age/sex	Presentation	Initial diagnosis	Time lapse (months)	Final diagnosis	Ref.
53/M	Diffuse joint effusions; clubbing	Rheumatoid arthritis	Not given	HOA secondary to end-stage cryptogenic cirrhosis, interstitial lung disease	[6]
54/M	Diffuse joint effusions; clubbing; positive RF and anti-CCP	Rheumatoid arthritis	Not given	Primary HOA and rheumatoid arthritis	[12]
30/M	Diffuse joint effusions; positive anti-CCP	Rheumatoid arthritis	12	HOA secondary to primary pulmonary hypertension	[13]
Not given	Diffuse joint effusions; positive RF and anti-CCP	Rheumatoid arthritis	1	HOA secondary to lung tumor and rheumatoid arthritis	[14]
55/M	Left elbow, bilateral wrist and ankle effusions; clubbing	Inflammatory arthritis	3	HOA secondary to small cell carcinoma	[15]
62/M	Bilateral ankle, wrist, right elbow effusions; clubbing	Inflammatory arthritis	4	HOA secondary to non small cell lung carcinoma	[15]
57/F	Clubbing	Inflammatory arthritis	8	HOA secondary to lung adenocarcinoma	[16]
57/M	Right knee effusion; clubbing; positive ANA and anti-Sm	Systemic lupus erythematous	18	HOA secondary to lung adenocarcinoma	[17]
47/F	Bilateral knee effusions; clubbing; positive ANA, anti-ds DNA, anti-SSA e anti-SSB	Systemic lupus erythematous	3	HOA secondary to lung adenocarcinoma	[18]
19/M	Diffuse joint effusions	Juvenile idiopathic arthritis	48	Pachydermoperiostosis	[19]
17/M	Diffuse joint effusions; clubbing	Juvenile idiopathic arthritis	Not given	HOA secondary to hepatopulmonary syndrome	[20]
50/M	Clubbing	Ankylosing spondylitis	36	Primary HOA (incomplete form) and ankylosing spondylitis	[21]
48/F	Diffuse joint effusions	Steroid myopathy	2	HOA secondary to chronic lung transplant rejection	[6]
70/F	Bilateral knee effusions	Infective arthritis	2	HOA secondary to small cell lung cancer	[22]
43/F	Bilateral knee and ankle effusions; clubbing	Lower extremity cellulitis	1	HOA secondary to past lung adenocarcinoma	[23]
66/M	Right knee effusion	Osteoarthritis of the knee	10	HOA secondary to lung adenocarcinoma	[24]

## Case presentation

A 77-year-old man was admitted to our Internal Medicine Department for fever, diarrhea, and a 1-month history of persistent polyarthralgia and prolonged morning stiffness at both ankles and knees. Almost 40 days before, the patient had been hospitalized for an exacerbation of chronic obstructive pulmonary disease (COPD), which was treated with a fluoroquinolone. Subsequently, he developed a *Clostridium Difficile* induced colitis, treated with oral metronidazole. The patient was a former port worker and had been a heavy smoker for 40 years. Past medical history included ischemic heart disease and arterial hypertension, for which he was taking anti-platelet and anti-hypertensive therapy.

On physical examination, he was febrile (37.9 °C) and had a heart rate of 95 beats/min and a blood pressure of 135/70 mmHg. His oxygen saturation was 96% on room air. His body mass index was 27 Kg/m^2^ and he reported a 5 Kg weight loss over the last 3 months. His ankles appeared clearly swollen, with reduced range of motion. There was a tense effusion in the left knee and a moderate joint tenderness in all limbs. Fingers and toes presented with a low grade digital clubbing, whose first appearance could not be precisely dated. Laboratory exams showed C-reactive protein (CRP) of 116.1 mg/L (normal values < 5 mg/L), erythrocyte sedimentation rate (ESR) of 90 mm/hr. (normal values < 35 mm/hr), white blood cells (WBC) of 11.860/mm^3^ (normal values between 4.000 and 11.000/mm^3^) and a rheumatoid factor of 56 U/mL (normal values < 20 U/ml). Blood and urine cultures were negative. The left knee synovial fluid aspiration showed an aseptic synovitis (8.226 WBC/hpf, with 90% neutrophils, while crystal detection, gram stain and cultures were negative). Moreover, *Clostridium Difficile* toxin in stool samples was still detectable.

Based on this presentation, a reactive arthritis due to a *Clostridium Difficile* induced colitis was suspected and oral vancomycin, oral prednisone, and painkillers were prescribed. Five days later, since fever, diarrhea, and polyarthralgia had apparently resolved, the patient was discharged, and a post-discharge visit was scheduled at the Rheumatology Office 1 month later.

**Fig. 1:**
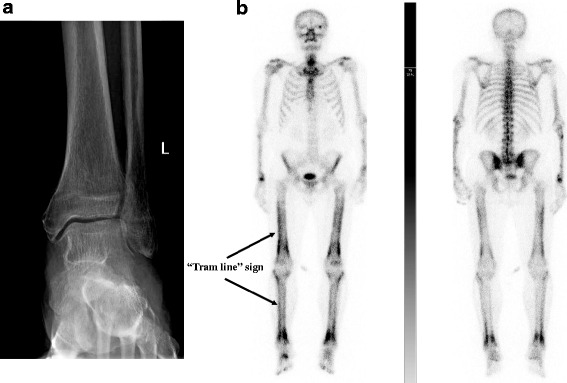
**a** Posteroanterior radiograph of the left ankle; (**b**) ^99m^Tc HMDP bone scintigraphy showing increased linear periosteal tracer uptake (“tram line” sign or “double stripe” sign)

**Fig. 2:**
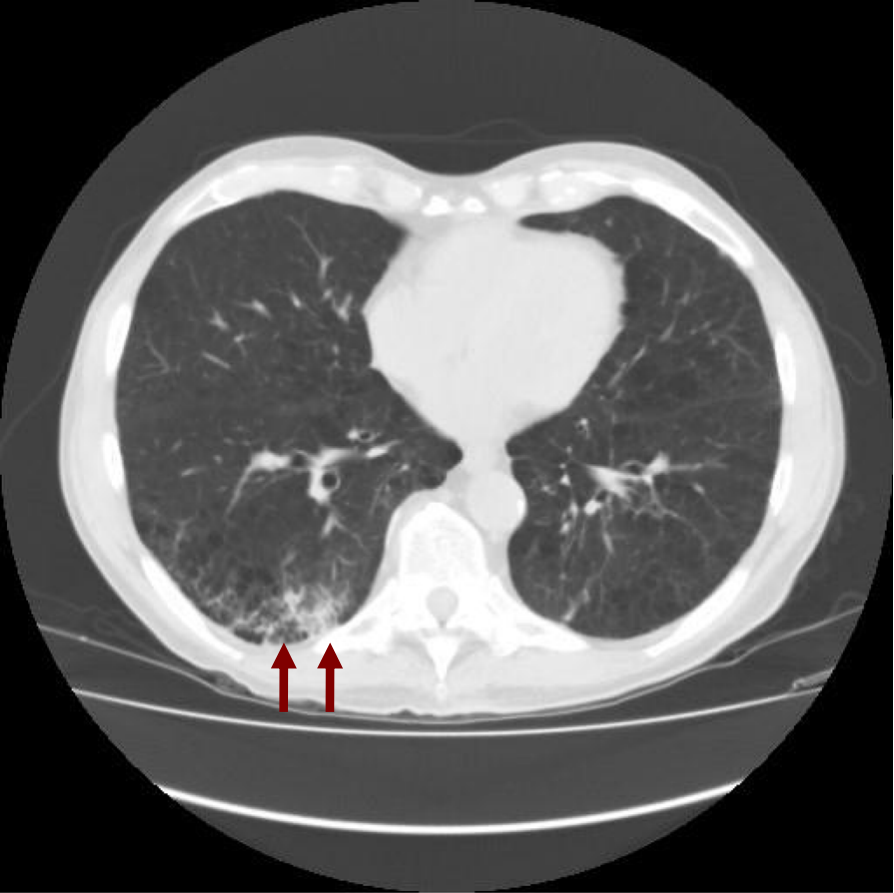
Chest CT showing lung emphysema with several pulmonary bullae and a lung infiltrate at the right lower lobe (red arrows)

When the patient came to the Rheumatology Office, he reported a relapse of polyarthralgia and joint tenderness while he was gradually reducing oral prednisone. He was slightly febrile (37.5 °C) but had denied relapse of diarrhea. On physical examination, his ankles were still swollen, while the left knee effusion had resolved. The fingers of both hands were also diffusely swollen and digital clubbing had worsened, with an increase of distal phalangeal/interphalangeal depth ratio (greater than 1.05). Lung auscultation revealed fine crackles at the base of the right lung. In laboratory exams, CRP was 101.1 mg/L, ESR was 99 mm/h and WBC was 14.380/mm^3^. Chest radiograph was unchanged as compared to that performed when the patient had the COPD exacerbation, revealing a possible patchy infiltrate at the base of the right lung, while radiograph of the ankles was inconclusive (Fig. 1a). At that stage, given the presence of a lung infiltrate and systemic inflammatory features (fever and elevated inflammatory markers), a relapse of right base pneumonia was suspected; thus, an empiric antibiotic therapy with a broad-spectrum penicillin and pain killers were prescribed. In addition, bone scintigraphy and chest computed tomography (CT) were promptly performed. The first revealed increased linear periosteal ^99^mTc-hydroxymethylene diphosphonate (HMDP) uptake, which is a typical sign of HOA (Fig. 1b). The second revealed a lung infiltrate with a partly ground-glass aspect (Fig. 2).

**Fig. 3:**
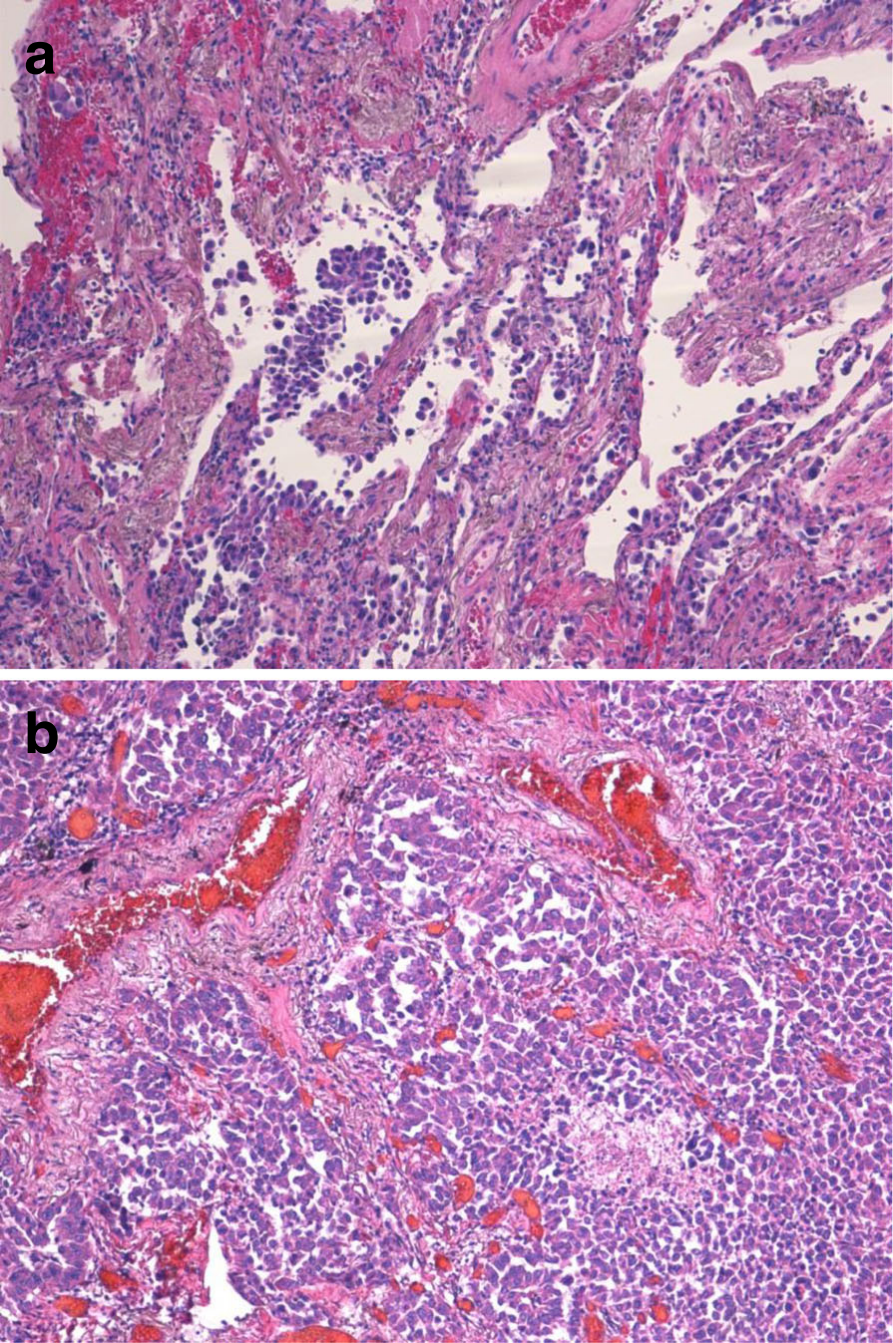
**a** Representative Hematoxylin and Eosin (H & E) stained section of transbronchial biopsy specimen, showing thickening of interalveolar septa and atypical pneumocytes proliferating along the surface of alveolar walls, compatible with a lepidic pattern adenocarcinoma of the lung (original magnification 10×); **b** Representative H&E stained section of lung autopsy, showing a lepidic predominant adenocarcinoma of the lung (original magnification 10×)

Owing to the high association between HOA and lung malignancies [3], the patient underwent a bronchoscopy with bronchoalveolar lavage (BAL) and transbronchial biopsies of the right lung infiltrate. Unfortunately, biopsies could not be performed because of the complexity in localizing the infiltrate under fluoroscopy. On the other hand, we were unable to schedule a CT-guided percutaneous biopsy or a diagnostic surgery because the patient’s general conditions dramatically worsened. He developed high fever (39.4 °C) and severe respiratory failure, which we ascribed to a severe bilateral pneumonia. During the hospitalization in a semi-intensive care unit, bronchoscopy was tried again and, finally, a transbronchial small biopsy could be performed. In the BAL, there were no malignant cells or infectious pathogens; likewise, blood and sputum cultures turned out negative. When the result of the transbronchial biopsy pathology examination (Fig. 3a) became available, showing a lepidic pattern adenocarcinoma, no specific therapy was prescribed due to the extreme gravity of the patient’s general conditions. Despite the use of noninvasive ventilation and extended spectrum antibiotic therapy, the patient died 4 days after the biopsy results and roughly 5 months after the initial clinical presentation. Autopsy confirmed the diagnosis of lepidic predominant adenocarcinoma of the right lung, associated with massive bilateral destructive pneumonia and severe emphysema with centrolobular pattern (Fig. 3b).

## Discussion

The first interpretation of the patient’s complaints was that of a reactive arthritis due to a *Clostridium Difficile* induced colitis. The incidence of *Clostridium Difficile* infections (CDI) is increasing worldwide, and fluoroquinolone use has been implicated in this context [4]. There are several reports of reactive arthritis due to CDI. Based on the literature, this disorder seems to affect young adults, mostly females. It usually develops 10 days after the CDI, and its initial presentation is a monoarthritis or oligoarthritis, most frequently involving the ankles and knees. The criteria for diagnosing a reactive arthritis associated with CDI include: (i) evidence of aseptic synovitis developing during or immediately after colitis; (ii) presence of toxin in stool samples; and (iii) absence of other causes [5]. Based on the initial presentation, which fitted all these criteria, our first hypothesis was that of a reactive arthritis.

Nevertheless, the patient’s age, his digital clubbing, and the lack of a good clinical response to steroid therapy led us to perform additional exams and finally to the diagnosis of HOA. Hypertrophic osteoartropathy, also known as Pierre Marie-Banberger syndrome, is a condition characterized by the triad of digital clubbing, periosteal reaction of long bones and painful tenderness of the limbs, especially in the lower extremities, sometimes with synovial non-inflammatory effusions of large joints [3]. When all three clinical features are simultaneously present, a complete form of HOA is diagnosed, but most often HOA presents as an incomplete form, with the possibility of digital clubbing being absent [6]. HOA can be a primary hereditary disease (pachydermoperiostosis) or, more commonly, it is secondary to several pathologic conditions, though mostly to lung malignancies (up to 90%) [3]. Digital clubbing, defined as a focal bulbous deformity of the tips of the digits, is one of the oldest clinical signs in medicine and it can be either isolated or associated with HOA. In addition, clubbing can be present in a wide variety of clinical conditions, including both neoplastic and non-neoplastic pulmonary diseases [7]. The quantification of phalangeal depth ratio (distal phalangeal/interphalangeal depth ratio) is one of the methods to measure the degree of digital clubbing and can be helpful to distinguish between patients with pulmonary malignancy and those with COPD. In fact, Baughman and colleagues have demonstrated that a phalangeal depth ratio exceeding 1.05 in a patient with COPD is frequently associated with the presence of bronchogenic carcinoma [7, 8].

Given that in our patient HOA was associated with worsening of digital clubbing and a chest infiltrate, a bronchoscopy was performed, showing a lepidic predominant lung adenocarcinoma, which was then confirmed by the autopsy. It has been shown that diagnosis of lepidic predominant lung adenocarcinoma (formerly known as non-mucinous bronchoalveolar lung cancer) [9] is sometimes quite challenging because it can exhibit radiological features suggestive of interstitial lung disease [10] or it can mimic a recurrent bacterial community acquired pneumonia [11]. In line with these reports, our case highlights that radiological evidence of interstitial lung disease or pneumonia does not rule out a pulmonary malignancy, but warrants further investigations in patients with a high clinical suspicion of lung cancer, such as in the case of HOA.

Diagnosis of HOA tends to be difficult since its clinical presentation can mimic that of other rheumatic diseases [6, 12–24] (Table 1), the first being rheumatoid arthritis [6, 12–14]. Furthermore, the presence of a rheumatic disease does not rule out a diagnosis of HOA, since HOA can also coexist with other rheumatic conditions [12, 14, 21] (Table 1). In addition, periosteal reaction, commonly considered to be the radiological hallmark of HOA [3], can also be present in other disorders [25], including some rheumatic diseases, such as polyarteritis nodosa [26], familial Mediterranean fever [27], Takayasu’s arteritis [28], psoriatic arthritis and reactive arthritis [29].

Periosteal reaction is the result of new bone deposition in response to different physical and chemical stimuli, and can develop either as a localized or as a systemic disease. In HOA, periosteal reaction tends to have a symmetric distribution and the earliest lesions are localized at the diaphysis of long bones of lower extremities, typically tibiae and fibulae [3]. Although HOA is most commonly detected with radiography, which can demonstrate periosteal reaction even in asymptomatic patients [3], in our case, this exam turned out to be inconclusive. By contrast, radionuclide bone scan with ^99^mTc-hydroxymethylene diphosphonate (HMDP), which is considered the most sensitive imaging modality for HOA detection and characterization [3], showed a symmetrically increased linear tracer uptake at the periosteal site (also known as the “tram line” sign), consistent with our clinical suspicion of HOA.

To our knowledge, we are the first to describe a case of HOA mimicking a reactive arthritis, although we cannot exclude the coexistence of both conditions. Some elements, such as the presence of an inflammatory synovial fluid in the left knee and the complete resolution of the effusion in this joint after 1 month of steroid therapy, might suggest that our patient had also suffered from a reactive arthritis secondary to a CDI. Moreover, the inflammatory condition promoted by the reactive arthritis might have contributed to the progression of HOA. In line with this hypothesis, HOA has been found to be associated with several inflammatory conditions such as sarcoidosis [30] and chronic infections [31–34]. Although pathogenesis of HOA is unclear, this condition has been linked to an increased production of prostaglandin E2 (PGE2). For instance, patients with both primary and secondary HOA have much higher urinary levels of PGE2 than healthy individuals [35–37]. PGE2 is a lipid mediator derived from arachidonic acid through the action of enzymes, including the ubiquitous tissue constitutive isoform cyclooxigenase (COX)-1 and the inflammatory or tumor-induced isoform COX-2 [38]. Increased levels of PGE2 might be responsible for secondary overexpression of vascular endothelial growth factor (VEGF), thus inducing neoangiogenesis, new bone formation and edema [36]. Therefore, VEGF inhibitors, such as a monoclonal anti-VEGF antibody and bisphosphonates [30, 39], as well as COX-2 inhibitors [35], have shown to induce relief of bone pain in patients with HOA.

With this in mind, apart from digital clubbing and the chest infiltrate, the other sign that raised our suspicion of an alternative diagnosis was the partial response to initial treatment. Treatment modalities for CDI induced reactive arthritis include NSAIDs, intra-articular corticosteroid injection or systemic corticosteroid therapy [40]. Generally, it is expected that two thirds of patients with reactive arthritis associated with CDI achieve a spontaneous recovery after 60 days from the onset of the colitis [41]. On the other hand, in the case of HOA, only curative treatment of the underlying cause can lead to the complete regression of periostosis and its corresponding symptoms [42].

## Conclusion

Our review of the literature confirmed that clinical presentation of HOA can be variable, frequently mimicking that of an inflammatory arthritis. Our case report reminds clinicians to be aware of HOA in elderly patients with a large joint arthritis, even in the presence of features suggestive of an alternative diagnosis, such as a reactive arthritis. For this reason, response to the initial treatment should be closely monitored in order to perform further diagnostic exams when it is only partial. This is particularly true when an occult neoplastic disease is suspected, as in the case of worsened digital clubbing. Furthermore, whenever a HOA is diagnosed, malignancy should be thoroughly searched, even if clinical and radiological exams could be just consistent with infectious or interstitial diseases.

## Abbreviations

ANA: Antinuclear antibodies
anti-CCP: Anti-cyclic citrullinated peptide antibodies
Anti-ds DNA: Anti-double stranded DNA antibodies
Anti-Sm: Anti-Smith antibodies
Anti-SSA and Anti-SSB: Anti-Sjogren’s syndrome-related antigen A and antigen B
BAL: Broncho-alveolar lavage
CDI: *Clostridium Difficile* infection
COX: Cyclooxigenase
CRP: C-reactive protein
CT: Computed tomography
ESR: Erythrocyte sedimentation rate
HMDP: ^99^mTc-hydroxymethylene diphosphonate
HOA: Hypertrophic osteoartropathy
hpf: High power field
NSAIDs: Nonsteroidal anti-inflammatory drugs
PGE2: Prostaglandin E2
RF: Rheumatoid factor
VEGF: Vascular endotelial growth factor
WBC: White blood cells
